# 

**Published:** 1884-01

**Authors:** 


### CONTENTS.

### EXTRACTS.

Treatment of Puerperal Septicæmia .......... 97
Danger of Poulticing Eyes .......... 107
Ratt e-Snake Poisoning Treated by Potassium Permanganate .......... 108
Treatment of Premature Baldness .......... 110
Useful Notes on Water .......... 111
The Transmission of Diphtheria from Children to Fowls .......... 111
Dr. Bartholow's Antiseptic Treatment of Typhoid Fever .......... 112
A Doctor's Diploma in Court .......... 113
Poisonous Ice Cream .......... 114
On Personal Precautions that may be Adopted by Medical Men whilst Attending Cases of Infectious Disease .......... 116
A New Treatment of Cancer .......... 117
Preventive Treatment of Mammary Disease .......... 119
A New Tune on an Old String .......... 120
A New Remedy for Diphtheria .......... 121
A Leech in the Larynx .......... 122
Treatment of Soft Chancres and of Buboes by Salicylic Acid .......... 122
Cough Mixture .......... 122

### EDITORIAL.

Practical Chemistry for Students and Physicians—Chapter Seventh .......... 123
Exercise Questions on Chapter Seventh .......... 124
The Water We Drink .......... 125
Remarkable Facts Relating to Insanity in the United States .......... 127

